# Supplementary material for: Lytic transglycosylases mitigate periplasmic crowding by degrading soluble cell wall turnover products
Source: eLife. 2022 Jan 24;11:e73178. doi: 10.7554/eLife.73178 (PMC8820737; doi:10.7554/eLife.73178)
Supplement: Figure 1—source data 1. [file elife-73178-fig1-data1.zip › UncroppedWesterns.pdf]

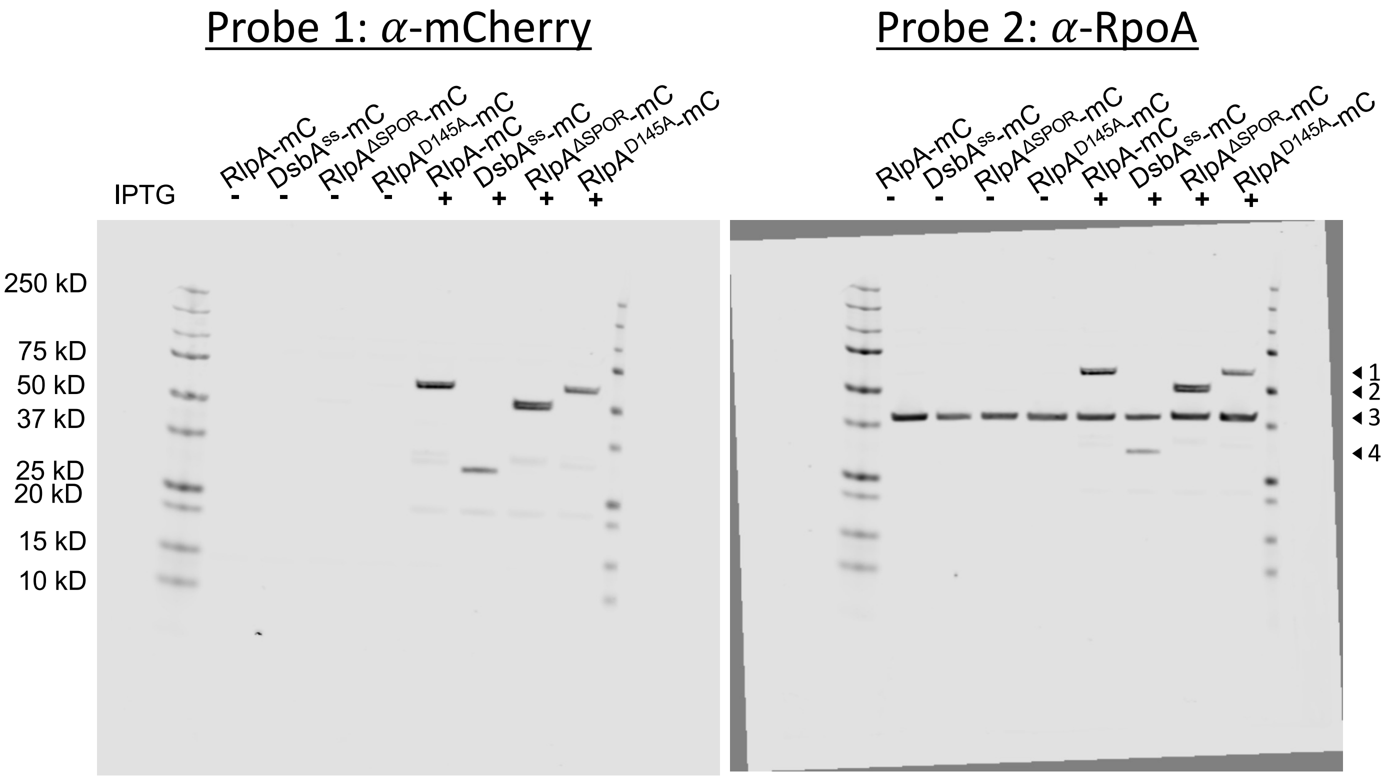

Major Band	Annotation	Approx. Predicted Size
1	Full length RlpA-mCherry fusion	55 kD
2	RlpA$^{\Delta SPOR}$-mCherry fusion	46 kD
3	RpoA	37 kD
4	DsbA^{ss}- mCherry<br>(Mature, secreted and signal peptide-cleaved)	27 kD
